# Gastric Electrical Stimulation for the Treatment of Obesity: From Entrainment to Bezoars—A Functional Review

**DOI:** 10.1155/2013/434706

**Published:** 2013-02-07

**Authors:** Martin P. Mintchev

**Affiliations:** ^1^Department of Electrical and Computer Engineering, University of Calgary, Calgary, AB, Canada T2N 1N4; ^2^Department of Surgery, University of Alberta, Edmonton, AB, Canada T6G 2B7

## Abstract

Growing worldwide obesity epidemic has prompted the development of two main treatment streams: (a) conservative approaches and (b) invasive techniques. However, only invasive surgical methods have delivered significant and sustainable benefits. Therefore, contemporary research exploration has focused on the development of minimally invasive gastric manipulation methods featuring a safe but reliable and long-term sustainable weight loss effect similar to the one delivered by bariatric surgeries. This antiobesity approach is based on placing external devices in the stomach ranging from electrodes for gastric electrical stimulation to temporary intraluminal bezoars for gastric volume displacement for a predetermined amount of time. The present paper examines the evolution of these techniques from invasively implantable units to completely noninvasive patient-controllable implements, from a functional, rather than from the traditional, parametric point of view. Comparative discussion over the available pilot and clinical studies related to gastric electrical stimulation outlines the promises and the fallacies of this concept as a reliable alternative anti-obesity strategy.

## 1. Introduction

### 1.1. Obesity as a World Wide Health Problem

The World Health Organization (WHO) announced recently that worldwide obesity has increased more than twice since 1980 [1]. WHO statistics outlined 1.5 billion overweight adults in 2008. A significant percentage of them were obese: nearly 300 million women and over 200 million men. An earlier report [2] revealed a diminishing initial age of obesity onset, with nearly 43 million overweight children under the age of five [1]. Even veterinary scientists have researched obesity in pets and its correlation with the obesity of their owners [3].

The WHO list of world's top 25 fattest countries covers all continents except Antarctica and sub-Saharan Africa [1, 4]. 65% of the world's population lives in countries where overweight and obesity kill more people than underweight and hunger. Moreover, obesity rates in the United States, Australia, and Canada increase faster than the overall worldwide rate [5]. Projections up to 2030 indicate that more than 36% of the population in the developed countries will be overweight and more than 22%—obese [6]. WHO prognosis for 2015 is that overweight adults will balloon to 2.3 billion, affecting both the developed and developing world [1, 2, 6].

Obesity is an important key factor for comorbidities and related mortality and is also increasingly considered as a type of cellular malnutrition and a noncommunicable disease [7]. WHO Global Strategy on Diet, Physical Activity and Health for 2008–2013 provides a roadmap to improve dietary and physical activity patterns at population level. Along with that, the global scientific community has been developing diverse treatment approaches.

### 1.2. Current Obesity Treatments

Contemporary obesity treatment approaches can be divided into two main streams: conservative, represented by diets [8–17], pharmacotherapy and/or behavior modification [18–22], and invasive, represented by bariatric surgery [23–30]; gastrointestinal electrical stimulation [31–38], and a variety of invasive endoscopic techniques, including intragastric bezoars and balloons [39–51]. 

The recently proposed temporary controllable pseudobezoar concept [52–58] can be considered a bridge between the two main obesity treatment streams—starting as a completely conservative, noninvasive approach—this therapy literally grows in the human stomach to an intragastric bezoar-based treatment, with the capability to be discontinued in a controllable noninvasive fashion. Attempts have been made to combine bezoar-based therapy with gastric electrical stimulation, but the evidence of efficient electrical stimulation from the mucosal side of the gastrointestinal tract remains anecdotal [52–55].

#### 1.2.1. Conservative Antiobesity Therapies

**Diets, Dietary Regimens, and Dietary Supplements**

As the most popular weight loss approach, diets and dietary regimens continue to exhibit an impressive growth. The current US public health standard for weight loss and/or chronic disease prevention is the combined low-fat (LF)/high-carbohydrate (HC) diet. A systematic review of more than ten randomized 6-month controlled trials concluded that low-carbohydrate/high-protein diets are equally or more effective than low-fat diets in terms of weight loss and cardiovascular disease risk for up to 1 year [8, 9]. Complementarily, it has been reported that vegetarian diets based on water and plant/fibrous foods rich in complex carbohydrates with low glycemic index increase satiety, lower the body mass index (BMI) and seem to be a reliable approach for the prevention of the obesity in children [10]. Similar effects were achieved via protein-rich diets, but not with dietary proteins [11]. Fiber-based dietary supplements, as well as their intragastric expandable subgroup, are often preferred when improved long-term sustainability of such diets is targeted [12].

Traditionally, the first step of standard obesity management is a combined treatment consisting of calorie-restricting diet, physical activity, and behavioural modifications [13, 14]. Due to the strong dependency of diets on individual particularities, personal dietetic management was recommended for this weight loss strategy [14]. For the purposes of effective counseling, objective weight loss predictors for individuals on specific dietary plans are under exploration [15].

Unfortunately, diets and dietary regimens have very limited potential in terms of sustainability: only 5% of the patients sustained their weight loss for more than a year or two [16]. Moreover, the short-term weight loss effectiveness of dietary approaches, combined or not with drugs, is not more than 10% reduction of the initial body weight [17]. When a more rigid parameter is considered, such as the percentage of excess weight loss (%EWL) and its long-term sustainability, a 5% reduction sustainable for more than a year would be considered a success [14, 16].

**Pharmacological Treatment**

Most of the antiobesity medications are adrenergic or serotonin agonists and invoke appetite suppression by affecting the central nervous system. Alternative group of drugs deactivates pancreatic and gastrointestinal lipases and blocks digestion and absorption of ingested fats. Currently, orlistat (Xenical, Roche Laboratories, Nutley, NJ, USA), phentermine (Lonamin, Medeva Pharmaceuticals, Sheldon Cir Bethlehem, PA, USA), and a combination of phentermine and topiramate (Osymia, Vivus Inc, Mountain Views, CA, USA) are the only three FDA-approved antiobesity drugs on the US market. Orlistat is a lipase inhibitor. Its application for a year is claimed to be effective for up to 30% of the patients, resulting in >5% weight loss. Most common side effects of orlistat are significant malabsorption of fat soluble vitamins, gastrointestinal problems, and liver malfunction [18]. Phentermine suppresses appetite as a sympathomimetic amine. Used alone, it leads to an 8–10% weight loss with a plateau at 3–6 months. Blood pressure elevation and disturbing heart rate changes are the most frequently reported side effects [19]. Phentermine combination with topiramate (an anticonvulsant, an antiepileptic drug itself) has been recently approved under the trade name Osymia. The long-term effects of this combination remain to be seen. The most popular appetite suppressant sibutramine has been recently withdrawn from the US market because of the increased risk of adverse cardiovascular events [20].

Approaches based on a combination of active substances which trigger several but different synergetic mechanisms represent an emerging direction in the pharmacological treatment of obesity. Therapeutics for migraine and seizure disorders, antidepressants, opioid antagonists, antiepileptic drugs, smoking cessation formulas, leptin, ghrelin, hormones with both glucose-regulatory and anorexigenic actions are only a small representative part of a wide spectrum of medications targeted as potentially effective antiobesity combinations [18, 21].

Generally, pharmacotherapy has a limited role in obesity treatment. Its efficacy is questionable due to the lack of reliable data for long-term adverse effects, short-period compliance, moderate and non-long-term sustainable weight loss, and most importantly, the significant risk of extreme and even life-threatening adverse side effects [22].

#### 1.2.2. Invasive Antiobesity Therapies

**Bariatric Surgeries and Lapbanding**

Compared to diets and pharmacotherapy, the effectiveness of weight loss surgery is long-term sustainable and can reversibly influence obesity-associated comorbidities [23]. Open vertical/horizontal gastroplasties and laparoscopic adjustable gastric banding contour the wide range of surgical weight loss techniques. They strongly influence gut hormone profiles [24] and modify gastrointestinal anatomy temporarily (gastric banding [25]) or irreversibly (Roux-en-Y bypass, biliopancreatic diversion, sleeve gastrectomy, etc. [25, 26]).

Clinical data indicate that Roux-en-Y gastric bypass and biliopancreatic diversion are the most successful techniques in terms of weight reduction sustainability, while sleeve gastrectomy results in the most rapid weight loss [27]. Although bariatric surgeries are hampered by short-term and long-term complications, morbidities and even mortalities, their benefits cannot be understated: rapid gastric emptying, decrease in appetite, favorable hormonal changes, and increase in peptide YY levels [25]. Mortality rate of bariatric surgery is between 0.1 and 2.0%, and the most common complications are anastomotic leak and ulcer [28]. 

Laparoscopic gastric banding (LAGB) is the most performed restrictive weight loss procedure. Unlike Roux-en-Y gastric bypass, LAGB does not cause nutritional deficiencies and can be adjusted during the weight loss process. It is safer (mortality rate of 0.05%), reversible, with reduced risk for anastomosis and much lighter complications, which are predominantly related to infections or erosion of the band into the stomach [25]. 

A novel endoluminal surgery performed entirely through the gastrointestinal (GI) tract by using flexible endoscopy is supposed to be a promising and less invasive alternative to open bariatric surgery [30].

The evidence clearly indicates that bariatric surgery is a more effective intervention for weight loss than nonsurgical options. Summarizing various studies, bariatric surgery led to a mean percent initial weight loss of over 20%, whereas the comparative nonsurgical groups lost only 1.5 to 5.5% of their initial weight. When reporting outcomes at two years, percent weight change ranged from a weight loss of 16% to 28.6% in the surgical groups, while the nonsurgical groups had actually gained weight with percent weight change ranging from 0.1 to 0.5% [23–30].

Of the available surgical options there is evidence that gastric bypass (GBP) is more effective for weight loss than vertical banded gastroplasty (VBG) and adjustable gastric banding (AGB). Greater weight loss has been reported following GBP than VBG with percent excess weight loss at one year ranging between 62.9% and 78.3% for GBP and ranging between 43% and 62.9% for VBG. Evidence shows laparoscopic isolated sleeve gastrectomy to be more effective than adjustable gastric banding with greater excess weight loss up to three years. Comparisons of GBP and laparoscopic sleeve gastrectomy (LSG), and of VBG and AGB produce similar results [23–30]. 

Price, invasiveness, reversibility, adjustability, complication rates, recovery period, abrupt changes in lifestyle, and eating habits [29] are important factors influencing patients' preferences for bariatric weight loss strategy.

**Endoscopic Weight Loss Therapies**

Currently the focus started shifting towards new, simple, safe, minimally traumatic, and reproducible nonsurgical approaches leading to a sustainable weight loss [39, 40]. Endoscopic treatments claim to respond to these expectations, offering a variety of approaches: gastric lumen reduction and procedures altering food absorption and space-occupying devices. Most of them have been recently proposed as noninvasive approaches offering effectiveness superior to pharmacotherapy which tend to reach that of the restrictive bariatric surgery while lowering its traditional risk profile [39, 40]. A variety of unique mechanisms (implantable intragastric prostheses, botulinum toxin, “butterfly” temporary pseudobezoars, etc.) associated with these classes of devices are being explored and utilized alone or in combination to activate multiple mechanisms for sustainable weight loss. Although remaining invasively positionable and removable, these devices represent a major and radical step towards sustainable but markedly less invasive treatments.

**Gastric Lumen Reduction (Transoral and Vertical Endoluminal Gastroplasty)**

Early experiences with endoluminal gastroplasty indicate its viability in obesity treatment [40, 41]. Novel endoscopic tools perform gastric plicating/partitioning with sutures (Bard EndoCinch Suturing System (C.R. Bard Inc., Murray Hill, New Jersey, USA), [42]), staples (TOGa (Satiety, Palo Alto, California, USA), [30]) or implanted devices (TERIS (Barosense, Inc., Menlo Park, California, USA), [43]). Transoral suturing for an endoluminal vertical gastroplasty is repeatable and provides significant weight loss in lower obesity categories: a decrease in BMI from 39.9 to 30.1 kg/m^2^ was reported 1 year after the procedure [27, 40]. It was suggested that the degree of weight loss in higher BMI categories may not be adequate [27]. Endoscopic staplers and implantable restriction devices are analogous to the adjustable gastric lapband in terms of mechanism and have shown similar weight loss and metabolic benefits [28].

Although there are options for the removal and/or volume modification of these implants due to necessity [43], the devices are generally permanent, which is an important limiting factor. All procedures are performed under general endotracheal anesthesia, although the techniques have the potential to be performed using conscious sedation [27]. Common patient complaints during recovery and prior to discharge have been reported to be nausea, vomiting, and abdominal discomfort [27].

**Food Malabsorption (Duodenojejunal Bypass Sleeve)**

The duodenojejunal bypass sleeve (DJBS) (EndoBarrier Gastrointestinal Liner; GI Dynamics Inc., MA, USA) is an endoscopically implantable impermeable polyethylene sleeve mimicking bariatric surgery. The results in terms of short-term weight loss and metabolic benefits for comorbid type 2 diabetes mellitus are promising. Complications because of device migration and anchor dislocation as well as continuous abdominal/epigastric pain and nausea during the first week after implantation were reported [28, 40, 44–46].

**Space-Occupying Devices (Intragastric Balloons)**

Intragastric balloons are the most studied [39] and the most commonly used endoluminal devices for non-surgical, totally reversible and repeatable treatment of obesity [13, 44]. The device is intended to reduce the food intake by reducing gastric volume from within the organ by distending inside the stomach and ultimately positioning itself in the fundus. Thus positioned, the balloon is effective if it is expanded to a minimum of 500 mL [47]. The mechanism of action is hypothesized to be a complex coactivity of multiple factors: mechanical gastric volume reduction, neuronal effects, changes in gastric motility, and hormonal profiles [39]. Balloons are increasingly used for the temporary treatment of obesity grades I and II. The method is also a preoperative treatment for obesity grade III [30, 41]. Intragastric balloon is an appropriate therapeutic tool for patients who aim to lose above 10% of their initial body weight and need to be assisted while adopting new eating and behavioral habits [17].

The most examined and widely used BioEnterics Intragastric Balloon (BIB) (Allergan Inc., Irvine, CA, USA) is usually inflated with 500 to 700 mL saline/methylene blue solution. The significant weight of the devices provokes abdominal pain or cramps, nausea, and vomiting [13, 48] which are reported to be the most common adverse events. Alternatively, air-filled polyurethane/silicone heliosphere balloon (HB) (Heliosphere BAG; Helioscopie, Vienne, France) has been designed (weighing only 30 g) to overcome the BIB's heaviness and the associated necessity for early removal. Both devices satisfactorily follow the recently formulated requirements in terms of smooth surface, soft/elastic building material, and radio-opacity [13–17]. Only a few clinical studies have aimed to compare the efficacy, tolerance, and safety of both designs. One of them [13] revealed similar efficacy 6 months after placement (weight loss of 12.8 ± 8 kg for HB and 14.1 ± 9 kg for BIB), as well as 12 months after retrieval. Based on separate BIB and HB studies, Lecumberri et al. [17] report favorable weight loss effectiveness for BIB: 9.3–13.4% for HB versus 17–20% for BIB. In all studies the therapy was supported by dietetic, physical, and behavioral therapies [13, 17, 48]. Nausea and vomiting during the first week after insertion are the most commonly reported side effects: 10–100% with HB versus 70–90% with BIB. The greater weight of BIB induces vomits and dehydration causing early removal in a higher percentage of cases compared to HB (20% for BiB versus 10% for HB). BIB has also higher rate of spontaneous deflations 2%–23% versus 1.6–6.3% for HB [13]. The HB's low pliancy (but large size) causes adverse problems during removal in about 1/3 part of the patients [17].

Few other types of intragastric balloons are also used: adjustable totally implantable intragastric prosthesis (ATIIP)-endogast, semistationary antral balloon (SAB), and silimed gastric balloon (SGB). The ATIIP-Endogast (Districlass Medical S.A., Corbas, France) is advantageous with its controllable option: its volume can be increased or decreased by the patient. Its migration is also significantly reduced, but problems with endoscopic insertion are still disturbing [49]. Other endoscopically positionable devices, such as the “Butterfly” and tubular membranes, are at experimental stages [41, 51].

Along with the reported side effects, implementation problems associated with HB manipulations that require anesthesia and pharmacological treatment are still hampering the wider application of balloons [17]. Gastric ulcerations, erosions and perforations, cardiac arrest, and even death are quite disturbing in respect to benefit/risk ratio of this method [50, 51].

All reported problems related to intragastric balloons prompted intensive research and development in two major directions: (a) utilizing mechanical rather than fluid-based expansion and (b) avoiding invasive placement and removal.

**Gastrointestinal Electrical Stimulation**

Electrical stimulation of the GI tract appears to be a promising therapy. Gastric electrical stimulation (GES) has been used for the treatment of obesity and drug refractory GI motility disorders. It has been reported that surgically implanted in the stomach, microelectronic devices (e.g., Transcend (Medtronic Transneuronix, Mount Arlington, NJ., USA), Tantulus (Dusseldorf, Germany)) delay gastric emptying, influence vagal efferents, and antral muscular contractions, and cause weight loss of 40% and 30%, respectively [31–33]. GES was supposed to be also a nonpharmacological approach to improve ghrelin secretion in several pathological conditions characterized by appetite disorders [21]. However, presently gastric electrodes have been implanted invasively from the serosal side of the stomach. Recently, endoscopic placement from the mucosal side was proposed as a feasible and safer alternative approach [34], but its effectiveness has not been adequately elucidated. Transcutaneous recharging and programming of implantable GES devices is considered a present-day industry standard [31–33].

Stimulation of the subdiaphragmatic sympathetic and vagal nerves is still being evaluated preclinically and clinically [31]. Several recent studies reported applications of intestinal electrical stimulation (IES) for the treatment of obesity [35–37]. All gastrointestinal electrical stimulation approaches will be discussed in greater detail in the present paper.

#### 1.2.3. Noninvasive Intraluminal Approaches: Bezoars

Bezoars are collections, concretions or foreign bodies that accumulate, coalesce, and are retained in gastrointestinal tract, most frequently within the stomach. Bezoars have also been located in the esophagus [59], small intestine, colon and rectum [60, 61]. Although the formation of intragastric bezoars is a rare medical phenomenon, it is well examined in terms of its origin, symptoms, effects, and treatment. The nature of the comprising materials defines several types of bezoars: phytobezoars (including diospyrobezoars), trichobezoars, pharmacobezoars, and lactobezoars.

The most common bezoars, phytobezoars, are formed by tangling fruit or vegetable fibers in the gastric lumen. Usually these bezoars are soft and can be composed by a great variety of indigestible phytofibers: grapefruit, apples, grapes, oranges, cherries, dried fruits, cabbage, celery, leeks, beets, bran, oats, peanuts, sesame or sunflower seeds, and so forth. The infrequent formation of diospyrobezoars (persimmon phytobezoars) is based on the polymerization of soluble fruit tannin assisted by gastric acid. The produced coagulum includes cellulose, hemicellulose, and protein and can reach a significant hardness [62]. 

Trichobezoars consist of plucked (trichotillomania) and ingested (trichophagia) hair, with or without trapped food material, fat, or medications [63]. Depending on the amount of tangled hair, they may alter drug metabolism due to medications' trapping within hair fibers [64]. The Rapunzel syndrome is a rare, complex modification of the trichobezoar, which crosses the pylorus and extends into the small intestines. Its accretion leads to iron-deficiency anaemia, frontal alopecia, abdominal pain (37%), nausea and vomiting (33.3%), complicated small bowel obstructions leading to perforations (25.9%), peritonitis (18.3%), and even death [65]. Patients have also presented with weight loss (7.4%), anorexia, hematemesis, and intussusception (7.4%) [65]. 

Pharmacobezoars are composed of medications and/or medication vehicles. They are formed by irregular utilization of medications due to, or combined with, abnormal gastric physiology. Reported cases describe a wide range of pharmacological products causing pharmacobezoars: aluminum hydroxide antacids, bulk-forming laxatives, cellulose-based enteric tablet coatings, clomipramine, vitamin/mineral supplements, extended-release medications, poorly digestible capsules, sucralfate, magnesium and calcium carbonates, potassium chloride, amitriptyline, verapamil, procainamide, and so forth [66]. Patients with pharmacobezoars are affected by two types of complications: the presence of the bezoar itself and the probable toxicity of the trapped medicine due to its altered properties or accumulated high concentrations. 

Lactobezoars are composed of undigested milk curd. Usually they are formed in the stomachs of infants or newborn babies between the first and fifth day–decade of life [67]. Cases of their passing through the gastrointestinal tract are also described [68]. Most often, lactobezoars' formation is provoked by calorie-dense formulas and causes gastric outlet obstruction [69].

Bezoars' etiology is based on ingestion of large amounts of indigestible material and various predisposing factors, postgastric surgery being one of the most important of them. Bezoar formation is commonly predisposed by postoperatively induced (a) atony, dysmotility, and delayed gastric emptying; (b) stenosis; (c) poor gastric mixing; (d) decreased secretion of acid and pepsin; (e) increased mucus production. Furthermore, partial gastrectomy, antrectomy, pyloroplasty, Roux-en-Y gastric bypass, and even laparoscopic adjustable gastric banding have been associated with phytobezoars, fungus balls, and bezoar formation in general [70]. 

Many of the bezoars are asymptomatic. Several classes of typically appearing symptoms are reported: vague epigastric discomfort (up to 80% of cases); nausea, vomiting and early satiety; weight loss and anorexia; bleeding, pain, and gastric outlet obstruction [65, 70]. In most cases symptoms are signals for pressure necrosis or gastric ulcers invoked by the bezoars. Small bowel bezoars (most often the Rapunzel syndrome) cause mechanical obstruction, usually in the hepatopancreatic ampulla. Additionally, iron-deficiency anaemia and frontal alopecia are typical accompanying conditions [71].

The most efficient and reliable method for bezoar diagnosis and resolution is endoscopy. Alternatively, upper gastrointestinal series with barium can detect gastric filling defects and and plain X-rays can determine the bezoar dimensions. Commonly, gastrointestinal bezoars are diagnosed late due to their infrequency and asymptomaticity. Bezoar treatments include conservative, endoscopic, and surgical approaches. Soft bezoars can be destroyed conservatively by dieting, enzymatic and chemical dissolution, therapy with prokinetic agents, or nasogastric lavage. Bezoar dissolution via liquid and nutritional diets is a slow process and is not preferred as a sole therapy. Fiber-containing bezoars can be partially influenced by proteolytic enzymes (papain, Viokase), while up to 85% of the phytobezoars can be successfully degraded enzymatically by cellulase formulations and the mucolytic agent N-acetylcysteine [72]. Aggressive lavage (water, coca-cola, and pineapple juice [73, 74]) and subsequent aspiration of the fragmented concretions is an appropriate approach for soft and permeable bezoars. For hard or large phytobezoars and trichobezoars, more aggressive therapies based on specialized techniques for mechanical disruption are preferred: laser pulsed water jets, mechanical or electrohydraulic lithotripsy. Nevertheless, severe complications such as esophageal perforation are always possible and endoscopic management is explicitly recommended for more symptomatic and obstructive bezoars. Most often symptomatic trichobezoars are resistant to standard gentle techniques and often require surgery (gastrotomy and enterotomy) more than other bezoar types [75–77]. Surgery lightens the patient's condition radically and rapidly but does not eliminate the reasons for bezoar formation. The risk of recurrence remains and gastric motility and emptying can even worsen.

Global quest for non-invasive, yet long-term sustainable antiobesity treatment methods has led to an impressive progress in intraluminal weight loss devices. They have been seriously improved in terms of technical precision, location, traumatism, anesthesia assistance, and so forth. The volume of the last generation intragastric balloons can be adjusted by the patient [49]. Despite these improvements, balloons are still inserted and removed endoscopically, and different levels of sedation and anesthesia are needed for both procedures. Responding to the global quest for weight loss novelties, several implementations were announced as novel approaches for gastric volume reduction based on ideas borrowed from the effect naturally occurring bezoars have on appetite: (a) “butterfly-” type bezoars [56], (b) expandable gastroretentive systems [57], and most recently, (c) controllable temporary pseudobezoars [52–55]. 

Hashiba et al. have reported experimental designs of artificial bezoars made of digestive-resistant polyethylene [78, 79]. More than 30 meters long strip, previously divided into loops of 40 cm, is folded in the middle. It is in a partially compacted configuration during the instrumental insertion in the gastric lumen. Inside the stomach the device expands sufficiently to be retained there for a long time without passing through the pylorus into the intestines. A subsequent modification of this device improved its compactness while inserting. It is implemented by a significantly shorter (3 m), but wider polyethylene flexible tube, and compressed over a plastic tube which is used as a guide and a pusher. It is deployed into the stomach after intraluminal endocopically controlled manipulations. Animal testing revealed effective weight loss over a several-month period [56]. Compared to balloons, this device seems to have several advantages: does not need to be inflated; has the opportunity for size adjustment; does not have valves to keep fluids; and does not require static positioning in the stomach. Unfortunately, the effectiveness of the device for obese patients has been referred to future studies [78].

 Expandable gastro-retentive therapeutic systems [57] have been designed primarily to control active substance release in the GI tract by gastric emptying delay. Such a system usually consists of an element which controls the active-compound release, as well as a polymer cover. The release controllability can be performed in different ways. One of them is via a substance which expands on contact with gastric juice. Long-time retainment in the stomach is achieved noninvasively due to an appropriate design and composition. The use of the device as an appetite/hunger suppressant is possible due to its expandability and its subsequent solid-food-like behavior. However, weight loss data obtained with this technique have not been reported. 

A novel temporary, controllable pseudobezoar weight loss concept was formulated recently [58, 80, 81]. It proposes the utilization of artificial, temporary pseudobezoars which is intended to combine the noninvasiveness of the conservative therapies with the long-term sustainable weight loss effect of the invasive antiobesity approaches. Pseudobezoars imitate the effect of phytobezoars [82] and trichobezoars [71] on weight loss. It is a well-known fact that bezoar formations induce anemia and wasting, resulting in appetite suppression and substantial weight loss [81]. The artificial bezoars are designed as orally ingested, intragastric, and self-expandable units. They consist of biocompatible, sac-like carrier filled with cellulose-based expandable fibers. They fit into standard gelatin capsules. Ingested with an abundant amount of water, they expand rapidly in the stomach thus forming nonnutritional volume in it. Postexpansion dimensions of pseudobezoars prevent their expulsion through the pylorus, and they are retained in the stomach for a controlled time period. Their regular intake reduces gastric volume similarly to the way intragastric balloons do, thus invoking constant feeling of satiety. Pilot and blind, placebo-controlled chronic human studies [58, 80, 81] have confirmed the viability of pseudobezoar approach as an alternative, noninvasive, safe, and effective weight control method. A 30-day therapy was reported, resulting in increased satiety, significant weight reduction, and insignificant side effects. Pseudobezoar retainment, collection, and clearance from the stomach were monitored sonographically. Their insertion and removal are totally noninvasive without any anesthesia. More importantly, the pseudobezoars are pH-controllable, being capable of spontaneously disintegrating in several hours if gastric pH is raised to above 6 by administering strong antacids.

Generally, however, combining artificially created gastric retentive bezoars with gastric electrical stimulation from the mucosal side of the stomach has been mentioned only parenthetically and has not been thoroughly explored.

## 2. Aim of the Present Paper

The aim of the present paper is to discuss in detail the promises and the fallacies of gastrointestinal electrical stimulation for the treatment of obesity and analyze the evolution of this technique from the initial approaches for disturbing gastric electrical entrainment to the present day attempts at electrical stimulation from the mucosal side of the stomach. In contrast with similar review articles on the subject, a functional rather than parametric classification of the various gastrointestinal stimulation methods has been suggested and pursued.

## 3. Types of Gastric Electrical Stimulation for the Treatment of Obesity

Gastric electrical activity (GEA) is a complex phenomenon resulting in gastric motility, which in turn leads to gastric emptying [83, 84]. Essentially, the stomach, in contrast to the heart, is an asynchronous pump. In other words, it is mechanically active only intermittently and not permanently. For this reason, gastric electrical activity is more complex than cardiac electrical activity. In essence, it has an omnipresent, periodic component, known as the electrical control activity (ECA) [85], which has a frequency of repetition in humans of only about 0.05 Hz or 3 cycles per minute (cpm). This activity originates somewhere in the proximal stomach, but no dedicated or anatomically modified pacemaker cells have been identified to clearly establish a pacemaker region similar to the sine node in the heart [86, 87]. In fact, the hypothesized pacemaker area of GEA changed several times over the years, with the improved sensitivity of our recording equipment [88]. Therefore, it has been suggested that the pacemaker cells are in fact distributed throughout the organ with an increased density in distal direction [89, 90], and the discovery of the interstitial cells of Cajal seems to confirm such suggestion [91]. Regardless, the presence of the periodic ECA is related to the opening of the sodium and the potassium channels on smooth muscle cellular level in a small circumferential band of cells detectable in the proximal corpus, and the resulting depolarization wave propagates distally with an increasing velocity [90], but this does not necessarily mean that immediate circumferential gastric contractility would follow. The latter occurs only if the cellular calcium channels open within the depolarized circumferential band of smooth muscle cells [92]. What makes gastric motility even more complex is that two types of calcium channels can open, the slow calcium channels, associated with what is known as type I electrical response activity (ERA-I), and the fast calcium channels, known as Type II Electrical Response Activity (ERA-II) or spike activity [93]. All these types of gastric electrical activities are interdependent, with ECA being the necessary but not sufficient condition for ERA of any type to occur and ERA-I being the necessary, but not sufficient condition for ERA-II to occur [93]. In other words, the omnipresent rhythmic 3 cpm sodium-potassium pump provides the synchronization of the overall gastric electrical wave propagating with an increasing velocity from the proximal corpus to the distal antrum, but it is the opening of the slow, and more importantly, the fast calcium channels, which is responsible for gastric motility resulting in the distal movement of gastric content and in eventual gastric emptying [94]. It is important to note that only ERA-II, or the spikes, has been associated with gastric contractions stronger than 0.025 N. Therefore, it can be said that only the presence of spikes is directly related to any significant content-propelling gastric motility. However, as we already pointed out, ERA-II, or the spikes, cannot occur without the presence of ERA-I, as it is superimposed on it, and both ERA-I and ERA-II cannot occur without the presence of ECA. In fact, ERA-I and ERA-II can only occur in complete synchronization with a 20-second ECA wave, but this does not mean that each and every ECA wave is accompanied by ERA. In the fasting state, the so-called migrating myoelectrical complex (MMC) is present, which practically can be split into four phases, a resting phase I, semiactive phase II (some ECA waves are accompanied by ERA, and some of the ERA waves contain both ERA-I and ERA-II), an active phase III (each and every ECA wave is accompanied by both types of ERA), and another semi-active phase IV as a precursor to another resting phase [95]. The overall periodicity of the MMC in humans varies individually but is in the range of 80–120 min, with the active phase III lasting 15–25 min [95]. With meals the MMC is broken, and the work of the stomach starts to resemble a semi-active phase of the MMC until it empties completely [96]. After gastric emptying, the fasting MMC pattern is restored [97]. However, the individual variability in both the fasting MMC and in the postprandial motility patterns renders this classification quite vague [98]. 

Based on the complex physiology of GEA and the functional results from applying gastrointestinal stimulation, seven major types of gastric electrical stimulation (GES) for the treatment of obesity can be identified. They are analyzed separately.

### 3.1. Disturbing Gastric Electrical Entrainment

Clearly, the entrainment of GEA, and particularly, of its ECA component from the proximal corpus to the distal antrum, is pivotal for the synchronization and the propagation of gastric contractions, which ultimately lead not only to the mixing and the grinding of the ingested food, but also to gastric emptying.

The concept of entraining gastric electrical activity to facilitate “proper” gastric motility is based on the hypothesis that electrical rhythm disturbances are the underlying reason for a variety of gastric motility disorders, including gastroparesis and possibly functional dyspepsia [99]. Thus, if gastric electrical signals are well synchronized in their propagation pattern (i.e., if they are entrained), the motility of the organ would be normal. The reversal of this logic, then, would mean that if gastric electrical signals are disentrained by disturbing electrically their normal synchronization, abnormalities in gastric emptying can be induced leading to its delay, and, respectively, to early satiety and weight loss [99, 100]. 

However, evidently, although the hypothesis of “correcting” gastric motility by entrainment, resulting in a treatment for gastroparesis, claimed initial speculative victories [101–104], it has never been verified in a serious clinical study [105]. Similarly, disturbing the entrainment of gastric electrical activity by retrograde pacing has been attempted [106–108] with reported success on small animal and human population samples, but a reliable clinical study involving statistically significant number of patients confirming these findings is still lacking. Not surprisingly, this technique did not lead to any FDA-approved medical device product for the treatment of obesity. Moreover, the entrainment approach itself did not lead to any clinical study demonstrating its utility nor to a medical device product for the treatment of obesity, functional dyspepsia, or dumping, the three major gastric motility disorders [38]. 

The reasons for the failure of these approaches might be numerous, but most likely their roots are related to the fact that gastric electrical desynchronizations and dysrhythmias might not be such pivotal factors in gastric dysmotility and disturbed gastric emptying. In fact, only several studies published reports about spontaneously existing intermittent “tachygastria” and “bradygastria” in humans, and their impact on gastric emptying remain quite unclear [109, 110]. Most likely, this impact is minimal to none, and gastric emptying still occurs despite their presence, something readily observed in dogs [111] and pigs [112]. To apply the reverse logic to this argument, intermittently disturbing a normally electrically coupled stomach would hardly lead to consistent, predictable, repeatable, controllable, and reliable delay of gastric emptying, unless the direction of propagation of all gastric electrical waves is disturbed leading to its complete propagation reversal from the antrum towards the corpus. In this latter case, however, vomiting in the subjects would inevitably be induced, and possible therapy for obesity would be prohibitive [113].

### 3.2. Directly Producing Retrograde Peristaltic Contractions

Direct production of gastric contractions by high-frequency, synchronized, and multichannel voltage or current trains has been the subject of intense investigation since first published in 1996 [114]. Numerous patents followed [115–118], and many scientific papers were published [32, 119–121]. Essentially, the method employs sets of electrode arrangements transverse to the gastric axis, which can be entirely or partially circumferential. When stimulated with high-frequency trains (>20 Hz, for durations >2 seconds), the smooth muscle tissue responds with strong circumferential contraction, which can be even lumen-occluding if amplitudes greater than 10 V (peak-to-peak) are utilized (in case of voltage-based stimulation [122]). The inclusion of multiple circumferential electrode set locations andthe synchronization of the administration of the pulse trains insequential fashion between these locations can propel gastric contentdistally, move it retrogradely, or simply shake it around on demandunder microprocessor control. This method has become known in the literature as neural gastrointestinal electrical stimulation (NGES) [123]. Naturally, the concept of producing controlled retrograde peristalsis using this technique has been tried for the treatment of obesity. Animal tests on a limited number of acute and chronic dogs as well as on humans [32, 119–123] produced exciting results. However, tissue accommodation and loss of smooth muscle contractile responsiveness have been reported after longer-term stimulation [33]. It has been hypothesized that this is related to the exhaustion of the local acetylcholine supply in the vicinity of the stimulating electrodes. This problem has been addressed by embedding a feedback-controlled system into the GES so that it is turned on “on demand”, rather than permanently, so that acetylcholine supply in the vicinity of the stimulating electrodes is naturally maintained by the body [33]. Unfortunately, clinical trials on human subjects using this technology in its original form are still lacking. However, a modification of that method resulted in a medical device known as the Diamond (Tantalus) System by Metacure (Dusseldorf, Germany). Clinical studies involving the device have indicated, however, that its efficacy is probably related to diabetic patients only [124–129]. The reasons for this limitation remain obscure [38].

### 3.3. Gastric Electrical Stimulation Not Affecting Entrainment, nor Contractions

Interestingly, at the end of the last century a new method for GES emerged, which was not based on entrainment of gastric slow waves nor was it based on directly producing circumferential contractions [130, 131]. Although initially it claimed an increased motility index in animal studies [130], subsequent research explicitly demonstrated that the technique of stimulating using trains of 12–15 cpm frequency does not affect gastric smooth muscles in any previously described way (entrainment of direct contractions). Numerous later studies, however, provided some evidence that this stimulation method exhibits antiemetic effect, which is an extremely burdening side effect for patients with severe gastroparesis [132–134]. Further placebo-controlled clinical studies, however, could not make that finding objective, and this type of therapy was approved by the FDA for the treatment of severe gastroparesis, but for humanitarian use only (Enterra Therapy, Medtronic, Minneapolis, MN, USA). To this day the mechanism of action of this method and how an antiemetic effect can be achieved with this type of electrical stimulation remain unknown [38, 135]. Nevertheless, a recent study confirmed that changing the electrode orientations of the Enterra device from longitudinal to transverse can lead to controlled gastric contractions [136]. Taking into account that the device is presently a two-channel unit, connecting it to produce antegrade invoked gastric peristalsis could be beneficial for accelerating gastric emptying in gastroparetic patients, but a retrograde alternative could perhaps be utilized for inducing early satiety and, respectively, to treat obesity. Such research, however, has not been yet reported, probably due to the fact that the ability of the Enterra device to produce circumferential gastric contractions has been reported only recently. 

Another interesting commercially available device for gastric electrical stimulation is the Transcend implantable pulse generator (IPG) by Medtronic Transneuronix (Mount Arlington, NJ., USA). A single lead is laparoscopically implanted at the area of the lesser curvature, and a stimulating current is applied with the following parameters: pulse amplitude = 10.0 mA, pulse width = 208 microseconds, burst “on” time = 2 seconds, burst “off” time = 3 seconds, and a burst rate = 40 Hz. Several initial studies in Europe [137, 138] landed the technology the CE certification mark, but in December 2005 Medtronic announced that the preliminary results of their Screened Health Assessment and Pacer Evaluation (SHAPE) trial using the Transcend device did not meet the efficacy endpoint of a difference in mean excess weight loss at one year [139]. In the absence of slow wave entrainment manipulation and of direct production of circumferential contractions, the eventual mechanism of action has not been unequivocally, adequately, and reliably elucidated [38]. Naturally, the device is not FDA-approved to this day. Thus, after the multiple clinical tests on limited number of animals and patients, but in the absence of placebo-controlled clinical data, the perspectives for this device look bleak as well. It should be mentioned, however, that it is very likely that similar to the Enterra Therapy by the same company, changing the configuration of the stimulating electrodes from longitudinal to transverse could possibly result in producing circumferential contractions, which can perhaps breathe a new future to this stimulation method, shifting it towards the NGES domain (see Section 3.2). However, to this day studies in that direction have not been reported. 

It should be mentioned that placebo-controlled trials involving implantable GES devices are difficult to justify ethically, and the very action of the surgical implantation of the device, albeit done laparoscopically, might be sufficient to cause prolonged weight loss for months. Perhaps this is the reason for some of the spectacularly exhibited lack of clinical reliability associated with these devices, resulting in the continuous approval rejections by the FDA for anything more than limited humanitarian use.

### 3.4. Intestinal Electrical Stimulation for the Treatment of Obesity

After the spectacular failures of some of the commercially available GES devices, which as a rule passed the CE certification threshold but failed the full-blown FDA approval in the USA, several research groups involved in their development now shifted their research scope towards intestinal electrical stimulation (IES). 

IES affects intestinal slow waves, contractions and transit through vagal, and cholinergic, and adrenergic pathways. Duodenal electrical stimulation (DES) and colon electrical stimulation (CES) are approaches that are still being explored. It has been claimed that DES successfully delays gastric emptying and reduces water intake [36]. The mechanisms underlying the inhibitory effect of DES on food intake and body weight may include a number of factors, of which delayed gastric emptying might be the most plausible, possibly due to an increased pressure towards the pylorus. Interestingly, if DES would prove to be a truly effective method to delay gastric emptying due to postpyloric manipulation, and thus indirectly reduce food intake, this could be a clear indicator that gastric emptying rate might be far more important for the process of food intake, compared to gastric accommodation. The latter would hardly be affected in any way by the postpyloric effect of DES. In a recent animal study CES demonstrated impressive delay of gastric emptying of solids (77%), reduced intestinal contractility and vagal activity, and increased sympathetic activity in the fasting state and reduction of 61% in food intake [37]. Reliable data on the long-term effectiveness of these treatments, double-blind, placebo-controlled studies, and reports on their sustainability and safety are still lacking [38].

The research pattern in this newly emerging area is very similar to its gastric predecessors—encouraging animal studies which as a rule are not placebo-controlled but claim effectiveness, while leaving the possible mechanism of action unclear [140–142], followed by several studies on a limited number of chronic patients, also not placebo-controlled and with unclear mechanism of action [36, 143]. Based on the less-than-glorious history of GES, such studies appear to be sufficient to attract the attention (and the investment) of major medical device manufacturers, which might lead to a CE certification, but whether an FDA approval would follow remains highly questionable.

### 3.5. Electrical Stimulation of the Vagal Nerve

The history of gastric vagal nerve electrical stimulation repeats to a large extent the developments surrounding the Transcend gastric electrical stimulator. After a burst of patent filings, multiple animal studies, as a rule not placebo-controlled and not involving statistically significant number of subjects, lead to chronic human trials [144–149], culminating with CE certification. The resulting device, Maestro Rechargeable System (EnteroMedics, St. Paul, MN, USA), has been considered for FDA approval, but to this day, such approval is still lacking.

Delivering high-frequency electrical stimuli to gastric vagal trunks (known as vagal block therapy or VBLOCK [150, 151]) has been hypothesized to cause intermittent intra-abdominal blocking of vagal nerve impulses, thus achieving weight loss in obese patients [151]. Very promising data have been reported, but as mentioned, the long-term impact of this technique, the long-term sustainability of the weight loss associated with it, the impact of the surgery itself on the reported short-term weight loss, the exact mechanism of action, and most importantly, its long-term safety have not been clearly elucidated and convincing. In addition, placebo-controlled clinical trials on statistically significant number of patients are still lacking. It should be mentioned that the vagal nerve might branch in the stomach, but it is connected to many other vital organs in the human body. The propagation length of the electrical stimuli applied to the gastric branches of the vagal nerve has not been studied, although a variety of stimuli durations, frequencies, and amplitudes have been tried, to conclude that durations of 90–150 s deliver the highest excessive weight loss (EWL) percentage [151, 152]. How far the electrical stimuli propagate, though? Do they reach the heart along the vagal pathway? Do they reach the brain? If they do, what is the long-term impact of this type of stimulation on these organs? These questions remain unanswered. Moreover, successful bariatric surgeries for the treatment of obesity are unequivocally associated with hormonal changes. Why have the clinical trials on any of the reported GES devices that claim successes in the treatment of obesity (including VBLOCK studies) not investigated the hormonal dynamics associated with the proposed therapy, and why have they not compared it to the well-established hormonal dynamics of the gold standard successful bariatric surgeries? The benefits of such safety and comparative studies would be unsurpassable. The result of all these unknowns became quite evident recently [153]—a serious, placebo-controlled, double-blind study could not demonstrate any efficacy associated with this type of GES.

### 3.6. Gastric Electrical Stimulation from the Mucosal Side

The separate listing of this type of GES is somewhat at odds with the overall functional classification concept of the present paper, simply because all previously discussed GES methods could be applied from the mucosal side, presumably delivering the same functional variability. One would think that these possibilities would create a family of alternative (and less invasive) GES techniques, since the electrode implantations could be done endoscopically, rather than laparoscopically. However, except several speculative publications reporting tests on limited number of animals and humans [154–160], this approach has not been very successful and has not resulted in a distinct certified marketable device in any major market jurisdiction. 

The reasons for that are quite clear-cut: (1) due to the significantly higher impedance of the gastric mucosa compared to the serosa, the eventual propagation of the electrical stimuli into the muscular and the neural networks in the stomach is probably quite limited, if existing at all [161], and (2) although the endoscopic implantation of the electrodes from the mucosal side might be easier, the exteriorization of the leads to connect them to the actual stimulator implant is problematic. One possible solution to the latter problem could be inductive coupling, but this might be realistic for very low-power devices that can possibly disturb gastric slow wave entrainment only, with all the limitations related to this method (see Section 3.1). However, the depth needed for the electrode implantation from the mucosal side in order to reach the circular muscle layer is such, that it is risky to puncture the stomach through, either during the operation procedure or after it, when the organ contracts either spontaneously or by invoking the contractions due to GES itself. Such adverse effect would be a medical emergency and would require immediate invasive surgery, rendering this technique even more dangerous than traditional bariatric procedures.

### 3.7. Bezoar-Based Gastric Electrical Stimulation

The idea of low-power, high-frequency (>50 Hz) voltages or currents stimulating gastric mechanoreceptors in order to increase the feeling of fullness and satiety was first tested in the former Soviet Union, and its pioneering comprehensive report was by Dr. Glushchuk in 1989 [162], but obviously, the implementation and testing preceded it by at least 5 years. The so-called “silver pill”, or SZhKT-4, is a medical device product certified for patient use in the Russian Federation, and is readily available for purchase for a modest price. In their recent D.Sc. dissertation Dr. Glushchuk dedicated a small subsection to the clinical applicability of the device [162, 163], but these claims were not independently confirmed, and for years the device has been advertised as the miracle pill which helped keeping the ageing members of the Politburo of the Central Committee of the Communist Party of the Soviet Union active. The device can produce programmable voltages of up to 10 V at programmable frequencies of 50 Hz or higher, but its current delivery capabilities are very limited for obvious reasons—battery capacity and capsule size. These stimulation parameters could be completely adequate for stimulating the mucosal mechanoreceptors in the gastrointestinal tract which interact with the brain to register the presence or the absence of food. The problem with the device, however, is related to the fact that it passes by some of these mechanoreceptors only once during its single-time transit through the gastrointestinal tract. Therefore, its effect would be momentous, or in other words, minimal to none. For a longer-lasting, and therefore, sustainable effect, frequent intermittent stimulation of the mucosal gastrointestinal receptors is needed.

Prolonged intermittent low-power electrical stimulation of gastric mucosal mechanoreceptors could be achieved in the stomach by combining GES with bezoar-based gastric retention technology [164]. Thus, one or, better yet, multiple gastric pseudobezoars having electrical stimulation capabilities could intermittently stimulate gastric mechanoreceptors both mechanically and electrically, inducing a controlled feeling of fullness and satiety in an entirely non-invasive fashion. After the battery of the device is exhausted, it falls apart and its constituent components safely exit the gastrointestinal tract due to natural peristalsis. Recently, a patent application has been filed to that extent [164]. However, although the purely mechanical presence of electrically passive pseudobezoars in the stomach has been already successfully tested on a limited number of humans [165, 166], the combined technique (gastric retentive pseudobezoar GES) has not yet been examined even in pilot studies, and its utility remains to be determined. 

## 4. Conclusion

In contrast to cardiac electrical stimulation, the history of GES is relatively long, but also relatively speculative and unsuccessful. Each and every new approach in the area starts enthusiastically, with encouraging reports on statistically insignificant number of acute animals, and reasonably quickly moving to chronic animals. At this point, the choice of an animal model, statistical significance of the animal population studied, and double-blind placebo control become pivotal for the future success of any given technique on chronic humans. However, exactly in this aspect the worldwide quest for optimizing the GES for the treatment of obesity is lacking. Hence, the actual limitations of a new GES method are usually discovered during the vigorous human clinical trials requested by the FDA, resulting in a prolonged medical device approval process or the lack of such approval altogether. This creates an aura of speculative uncertainty over the utilization of GES for the treatment of obesity, and a lot of serious academic and clinical work is needed to alleviate it. 

Of the seven distinct methods for GES classified on the basis of their functionality, two hold the greatest promise: (1) the technique associated with direct controlled production of retrograde gastric peristalsis (see Section 3.2); and (2) the combined bezoar-GES technique, which is in its infancy (see Section 3.7). Which of these methods would be more beneficial for the treatment of obesity in a broader segment of the ever-growing patient population can be determined only comprehensive, long-term, and placebo-controlled clinical trials on a significant number of patients. Unfortunately, such reports are presently lacking in all of the listed techniques. However, the first method has been already the subject of some clinical investigations, while the latter has been reported as an idea only.
